# Does lateral column lengthening reach the ankle? Lateral calcaneal lengthening for flatfoot and medial talar dome osteochondral lesions: A weight‐bearing CT 3D modelling analysis

**DOI:** 10.1002/jeo2.70915

**Published:** 2026-09-21

**Authors:** Marco Di Ponte, Silvio Caravelli, Giammarco Gardini, Claudia Capellini, Claudio Belvedere, Alberto Leardini, Carlo Capodagli, Victor Valderrabano, Cesar De Cesar Netto, Massimiliano Mosca

**Affiliations:** ^1^ IRCCS Istituto Ortopedico Rizzoli Bentivoglio Unit Bologna Italy; ^2^ Department of Biomedical and Neuromotor Sciences (DIBINEM) University of Bologna Bologna Italy; ^3^ Alma Mater Studiorum University of Bologna Bologna Italy; ^4^ IRCCS Istituto Ortopedico Rizzoli Movement Analysis and Functional Evaluation Laboratory Bologna Italy; ^5^ Swiss Ortho Center Basel Switzerland; ^6^ Faculty of Medicine University of Basel Basel Switzerland; ^7^ Department of Orthopaedic Surgery, Foot and Ankle Division Duke University School of Medicine Durham North Carolina USA

**Keywords:** distance mapping, patient‐specific, peritalar subluxation, progressive collapsing foot deformity, surgical planning

## Abstract

**Purpose:**

Osteochondral lesions (OCL) of the medial talar dome are frequently observed in progressive collapsing foot deformity (PCFD), and hindfoot malalignment is believed to influence their natural history. It is not established whether, or to what extent, a realignment osteotomy modifies the articular relationship at the site of such a lesion, nor is there quantitative guidance on the size of opening wedge to be selected.

**Methods:**

Starting from a severe PCFD case analysed via weight‐bearing computed tomography, a 3D model was created using segmentation software. In the model, a lateral calcaneal lengthening osteotomy (LCLOT) was performed 15 mm proximally to the calcaneocuboid joint: Evans Technique. Three standard LCLOT‐wedges (8–10–12 mm) were virtually applied at the osteotomy site.

**Results:**

The 3D rotation of the anterior osteotomy stump with 8 mm wedge implied, with respect to the original talar position, 6.6° of dorsiflexion, 2.9° of inversion and 10.9° of external rotation; corresponding values for the ten and twelve mm wedges were 8.6°, 5.9° and 12.6°, and 14.2°, 10.2° and 22.2°. Further distance map analysis of the calcaneal posterior facet revealed a distancing effect on the posterolateral zone of the posterior subtalar joint with each wedge size. Additionally, the distance maps of the tibiotalar joint, where a concomitant medial osteochondral lesion of the talar dome was present, showed an increase in inter‐surface distance between the medial talar zone and the mortise. This increase was progressively greater with the use of larger LCLOT wedges.

**Conclusions:**

The model confirms that Evans LCLOT produces a triplanar deformity correction, increasing the inter‐surface distance at the anteromedial tibiotalar joint adjacent to the medial OCL. Patient‐specific modelling may therefore inform preoperative planning when a talar dome OCL coexists with PCFD.

**Level of Evidence:**

Level V, preclinical simulation study.

## Graphical abstract

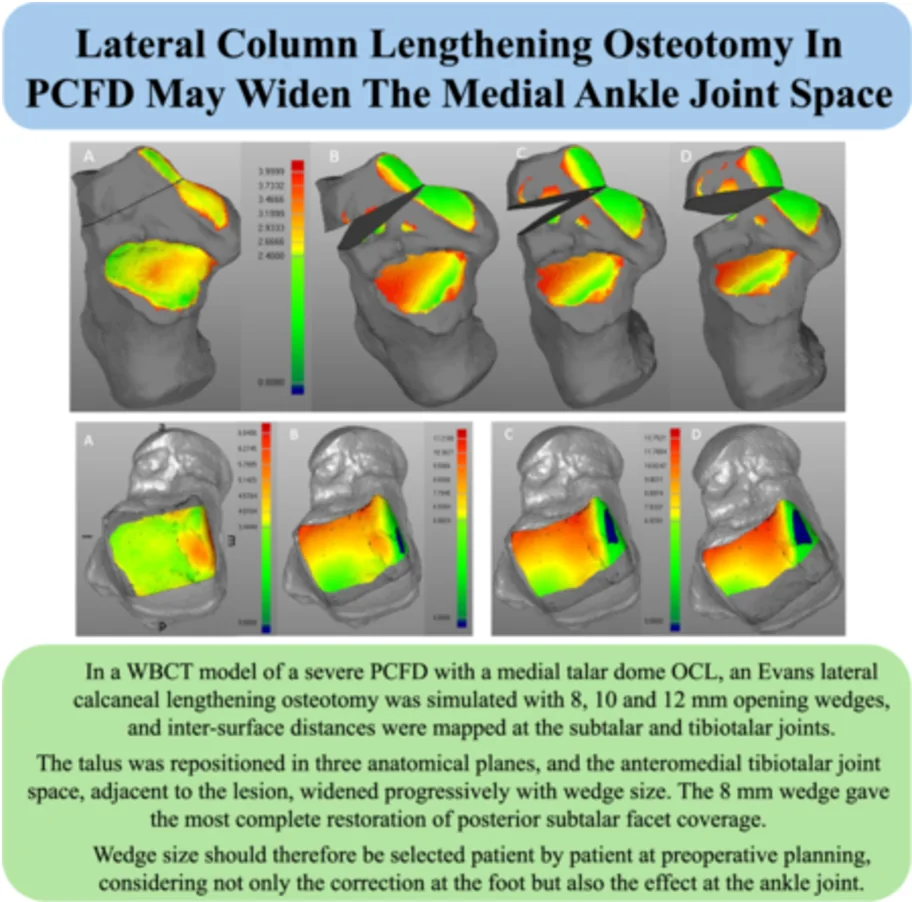

**Abbreviations**
LCL: lateral calcaneal lengthening
MDCO: medial displacement calcaneal osteotomy
OCL: osteochondral lesions
PCFD: progressive collapsing foot deformity
PTT: posterior tibial tendon
SMOT: supramalleolar osteotomies
WBCT: weight‐bearing computed tomography

## INTRODUCTION

Progressive collapsing foot deformity (PCFD) is a complex 3‐dimensional deformity with varying degrees of hindfoot valgus (Class A deformity), forefoot abduction (Class B deformity), arch collapse (Class C deformity) and peritalar subluxation (Class D deformity), which clinically results in pain and functional limitations [6, 7]. When a PCFD coexists with an osteochondral lesion (OCL) of the medial talar dome, the surgeon faces a specific and unresolved problem: whether realignment of the hindfoot alone is able to modify the articular environment of the lesion, or whether the lesion must be addressed as a separate procedure [8]. Biomechanical experiments have demonstrated that in valgus and pronated hindfoot the maximum pressure is usually located on the lateral talar border, however the hindfoot alignment per se does not correlate with the location of talar OCLs, and therefore OCLs may also occur on the medial talar border [2, 19]. During loading, cyclical compression and distraction of the cartilage, forces fluid into the microfractured subchondral bone, leading to a localised high increased flow and pressure of fluid in the subchondral bone. For this reason, malalignment of the ankle joint may aggravate this process by increasing the local pressure in specific locations of the ankle. In fact, medial displacement calcaneal osteotomy (MDCO) can unload specific tibio talar compartments, changing the loading axis at the level of the OCLs [9, 14]. Whether an Evans‐type lateral calcaneal lengthening (LCL) produces a comparable change at the medial tibiotalar joint has not been quantified, and no quantitative criterion exists for selecting the size of the opening wedge, which is currently chosen intraoperatively on clinical impression. Several surgical techniques have been proposed for PCFD, often without biomechanical validation [15], but recent advances in weight‐bearing computed tomography (WBCT) and computational modelling now enable personalised 3D weight‐bearing skeletal models to simulate and evaluate surgical interventions [10]. Combined with rapid prototyping technologies, these tools offer new opportunities for patient‐specific surgical planning and biomechanical assessment, although accurate and clinically reliable simulations remain challenging [11, 13, 20].

It was hypothesised that evans‐type LCL osteotomy would reposition the talus in all three anatomical planes, and that the resulting change in inter‐surface distance would be wedge‐size dependent and would differ between the tibiotalar and the posterior subtalar joint.

## METHODS

This preclinical, patient‐specific simulation study (Level V) was conducted on a single WBCT dataset, with the size of the opening wedge as the sole independent variable. The study was approved by local Ethics Committee, and the patient gave written informed consent in accordance with the Declaration of Helsinki.

The characterisation and visualisation of the distance distribution between the articulating surfaces of the bones in the foot and ankle complex was based on a distance mapping technique. The distance mapping algorithm (Geomagic Control™) computes the distance distribution between any two opposing articular surfaces by sampling the surface of a test object (bone 1) and identifying the corresponding minimum distance to a reference surface (bone 2). The colour‐coded distance maps can be read similar to topographic maps, being represented by a spectrum of 23 colours with each colour representing a different distance range to a reference surface. Green areas represent close approximations, whereas red colours show large distances between the two surfaces. Blue colours represent penetrating surfaces (negative distances), which can be the result of systematic errors of the image rendering process [1].

A 48‐year‐old patient presented with advanced PCFD and mechanical symptoms, including pain reproduced by weight‐bearing at the sinus tarsi attributable to bony or soft‐tissue impingement, along the posterior tibial tendon (PTT), and deep medial ankle pain. The patient had a history of worsening of pain following an ankle sprain. A weight‐bearing CT scan in single‐stance upright posture (OnSight 3D Extremity System®) was performed, revealing a subcentimetric OCL of the middle medial talar dome. A DICOM file of 960 CT images at 0.26 mm distance was exported, and a 3D mesh for the ankle and foot bones was generated automatically by segmentation software (Mimics® Innovation Suite version 22.0 ‐ Materialise), based on a pre‐set mask derived from Hounsfield units relating to cortical bone. When generating 3D bone models, an overall error of 0.26 mm must be considered, which corresponds to the slice thickness in the medical images. All measurements were carried out automatically and are reproducible.

The original overall skeletal alignment of the 3D computer model was modified by simulating the Evans‐type LCL osteotomy using the standard 8‐, 10‐, and 12‐mm opening wedges. In the anatomical reference frame of the foot, the osteotomy was virtually performed at about 15 mm posteriorly from the calcaneocuboid joint by targeting the sulcus between the middle and the anterior subtalar articular facets, orthogonal to the transverse plane of the foot. In the 3D model the hinge about which rotation of the posterior part of the calcaneus was performed, was calculated as the tangent to the 3D profile generated by the intersection of the calcaneal medial surface and the virtual cutting plane. The osteotomy plane showed a mean intra‐operator variability of ±1° over three repetitions with respect to the proximal‐distal axis of the anatomical reference frame of the foot. The 8, 10 and 12 mm openings were simulated on the basis of the distance between the opposing osteotomy walls, computed automatically along an axis lying in a plane perpendicular to the hinge and tangent to the osteotomy line on the lateral cortical surface of the calcaneal model. To assess the effect of this sequential lengthening of the lateral column, the talus was accommodated according to the best‐fit alignment on the calcaneal surface, minimising the root‐mean‐square surface‐to‐surface deviation.

## RESULTS

**Figure 1:**
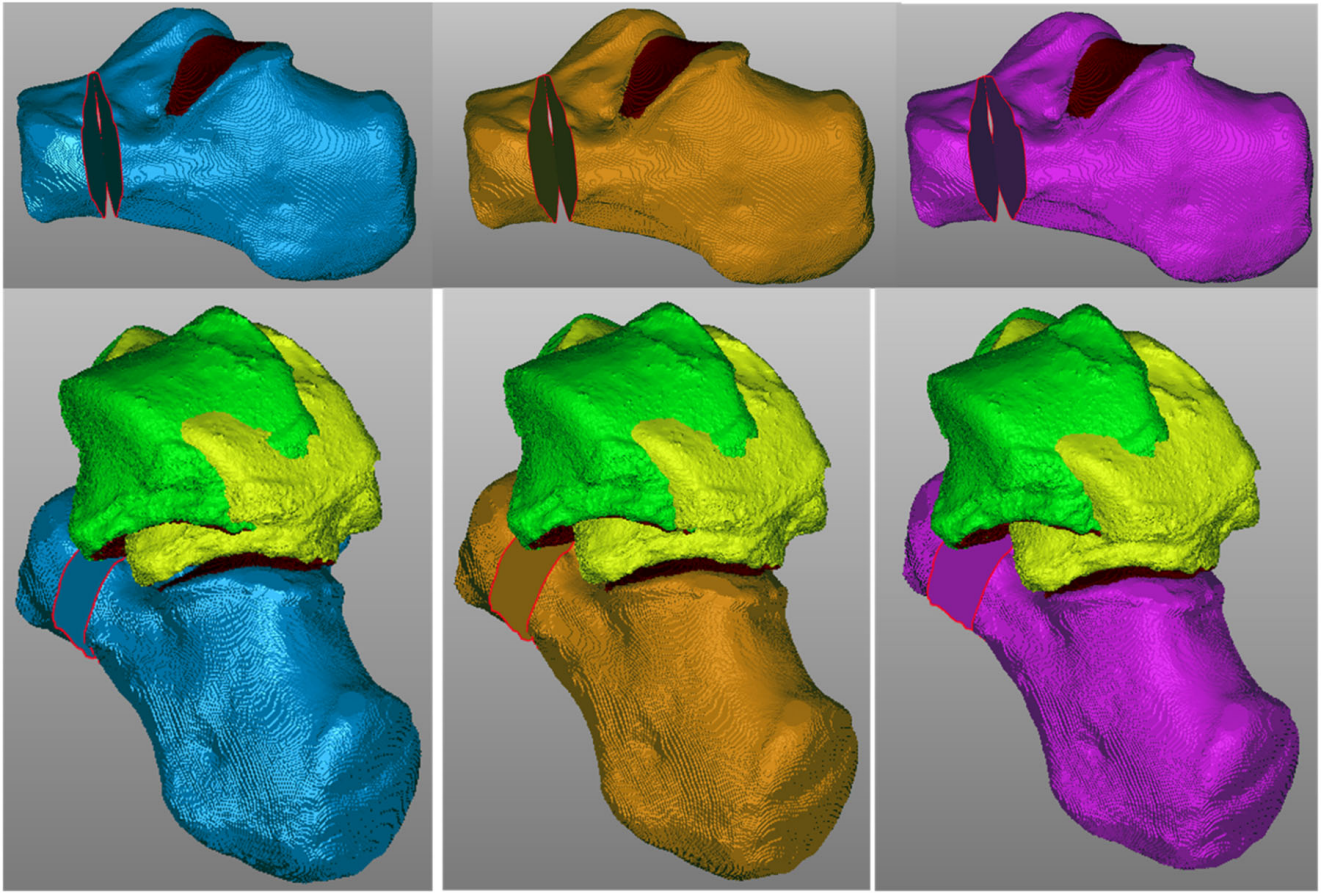
Evans‐type lateral calcaneal lengthening (LCL) osteotomy with 8 mm (left—blue), 10 mm (mid—orange), and 12 mm (right—purple) opening wedge. The green talus represents the initial position of the bone, the yellow one represents the post‐simulation configuration. The different colour representations are intended solely to facilitate the identification and distinction of the individual bones.

The three wedges were applied to the 3D bone model, which corresponded to 18°, 22.5°, and 27.7° of rotation of the anterior osteotomy stump on the hinge (Figure 1).

To assess the effect of this sequential lengthening of the lateral column, the talus was accommodated according to the best‐fit alignment on the calcaneal surface. Distance‐map‐analysis (‘3D compare’ tool of Geomagic® Control, 3D Systems) was performed between the calcaneus and the talus at each of the three lengthening steps and between the talar dome and the tibio‐fibular mortise. The 3D rotation of the anterior osteotomy block with the 8 mm wedge and the subsequent best‐fit alignment, resulted in 6.6° of dorsiflexion, 2.9° of inversion and 10.9° of external rotation about the longitudinal axis of the talus, with respect to the original talar position. The reference system used for this evaluation was the talar anatomical frame described by Conconi et al. [3]. Corresponding values for the 10‐ and 12‐mm wedges were 8.6°, 5.9°, 12.6° and 14.2°, 10.2°, 22.2° (Table 1).

**Table 1:** Degrees of talar repositioning relative to the original talar position following evans lateral calcaneal lengthening (LCL) osteotomy with 8‐, 10‐ and 12‐mm wedges.

Wedge size (mm)	Dorsiflexion (°)	Inversion (°)	External rotation (°)
8	6.6	2.9	10.9
10	8.6	5.9	12.6
12	14.2	10.2	22.2

**Figure 2:**
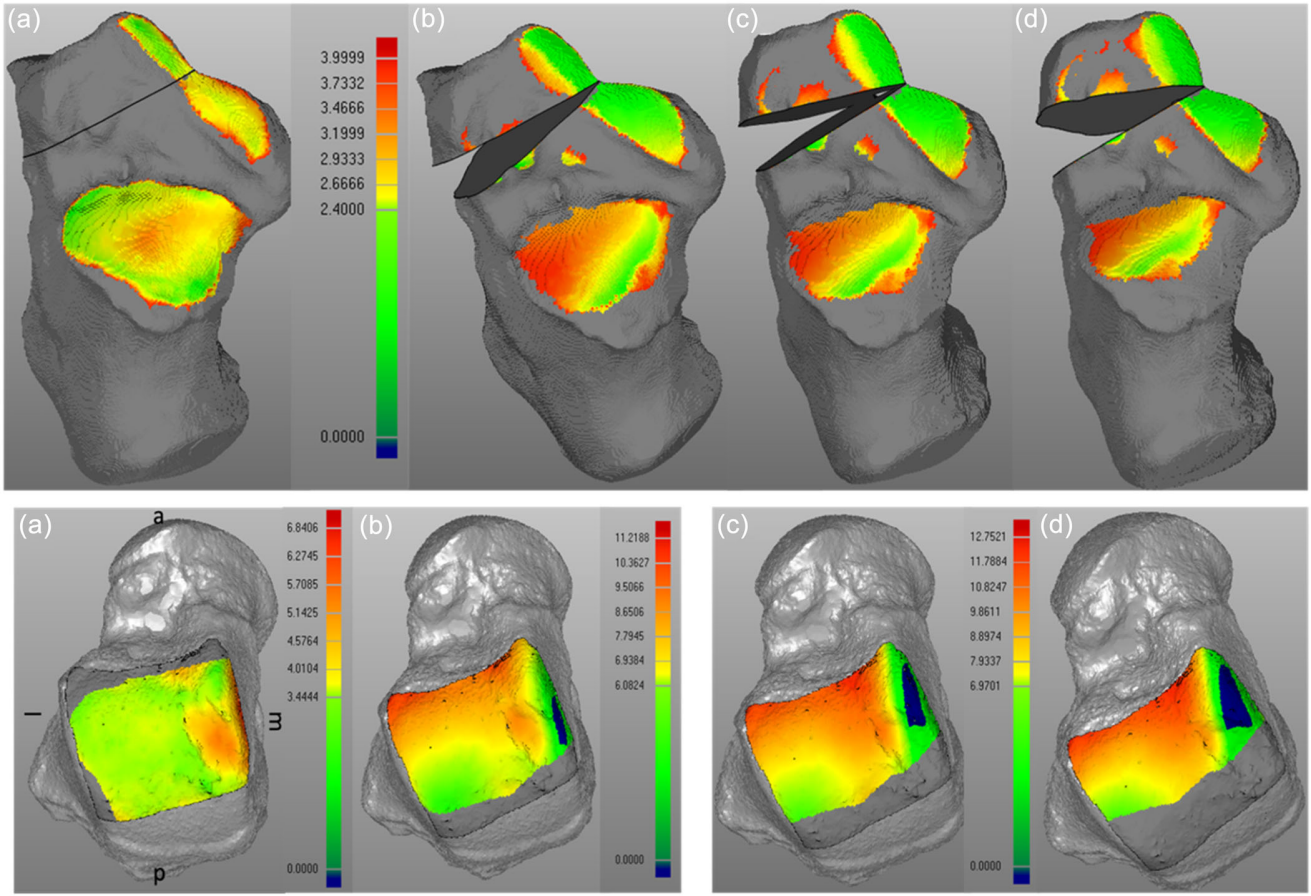
Distance mapping of the posterior subtalar facet (upper row) and talar surface/medial dome (lower row) in neutral condition (a), after 8 mm wedge (b), 10 mm wedge (c), 12 mm wedge (d). Each panel is rendered on its own colour scale: the distance between the joint surfaces was represented by a colour gradient green denotes close approximation, warmer colours increasing surface‐to‐surface distance, blue negative values from surface interpenetration (a rendering artefact).

Further distance map analysis of the calcaneal posterior facet revealed an increase of joint space laterally at the posterior subtalar joint along with a reduction of the surface‐to‐surface distance at the anterior and mid subtalar joint. Moreover, distance map analysis of the tibio talar joint shows an increase of the joint space anteromedially at the tibiotalar joint along with a reduction of the surface‐to‐surface distance at the posterolateral corner (Figure 2).

## DISCUSSION

The most important finding of the present study was that Evans‐type LCL not only corrected the PCFD but also repositioned the talus in all three anatomical planes, increasing, the inter‐surface distance at the anteromedial aspect of the tibiotalar joint, adjacent to the medial talar dome OCL, a condition frequently associated with this deformity.

The assessment of three‐dimensional deformity using WBCT in patients with PCFD has significantly advanced over the past decade. This imaging method has revealed peritalar subluxation, showing a reduction in the articular surface contact within the subtalar joint and a marked increase in the distance at the posterolateral angle of its posterior facet [1]. Following Evans‐type LCL, the distance map analysis of the calcaneal posterior facet revealed an increase in distance at the posterolateral angle of the posterior subtalar joint and a reduction of peritalar subluxation to a greater extent with the 8 mm wedge. This could suggest a reduced articular proximity at the medial zone, progressively greater with larger wedges. Therefore, realignment procedures might help reduce articular contact at the ankle joint sufficiently to relieve symptoms of mechanical impingement, although this cannot be established from the present geometric data and remains a hypothesis to be tested clinically. A potential advantage of this approach could be the possibility of addressing two pathologies with a single procedure in selected cases. Based on this theoretical framework, in a clinical study involving eight patients with previous OATS failure for medial OCL, supramalleolar osteotomies (SMOT) or MDCO procedures were performed without revision surgery of the OCL; these patients demonstrated a beneficial effect in terms of reduced subchondral cyst volume and improved clinical symptoms [14].

In medial talar OCLs, the deep deltoid ligament inserts more broadly and more proximally on the talus, and its fibres may be continuous with the osteochondral fragment [5, 17]. Chronic repetitive stress on this ligament, as occurs in PCFD, could therefore contribute to the development of medial talar dome defects [12, 18], and may also affect the deltoid branch of the artery of the sinus tarsi, which supplies the medial periosteal surface of the talar body [16]. From a clinical standpoint, the 8 mm wedge represented the most advantageous option for this patient: it restored posterior subtalar facet coverage most completely, whereas larger wedges overcorrected that relationship and carry a recognised risk of lateral column overload [4, 5].

The procedure outlined here presents limitations in terms of repeatability and reproducibility. Being fundamentally a geometric method based on the interface of selected areas, the critical factor lies in the ability to identify the same regions of interest in successive measurements and/or by different operators. Since no soft‐tissue constraint was modelled, the talar position at each step depended on the best‐fit criterion, which assumes maximal articular congruency; the reported rotations are thus generated by this criterion, and a different one could yield different values. Experimental tests conducted in two sessions revealed differences in the obtained rotations, which nonetheless remained consistent around the three axes. Among limitations of this 3D simulation is the absence of soft‐tissue modelling. In particular, the influence of muscles, tendons, and ligaments, structures that can modify and constrain the achievable angular correction, was not taken into consideration. Finally, the model is based on a single patient and has not been validated against postoperative weight‐bearing imaging or clinical and radiological follow‐up.

## CONCLUSION

Based on this study, the evans‐type LCL osteotomy effectively achieves triplanar correction of PCFD, increasing the inter‐surface distance at the anteromedial tibiotalar joint, adjacent to the medial OCL of the talus, by an amount dependent on wedge size, whereas the greatest restoration of posterior subtalar facet coverage was obtained with the smallest wedge. The increasing wedge size resulted in a progressively greater increase in this distance, highlighting its potential for relieving mechanical symptoms and addressing medial talus OCLs, although larger wedges must be balanced against the recognised risk of lateral column overload. The present entire original procedure illustrates how personalised 3D modelling of the foot and ankle in weight‐bearing may support preoperative planning when correcting advanced PCFD.

## AUTHOR CONTRIBUTIONS

Marco Di Ponte and Silvio Caravelli conceived and designed the study. Claudia Capellini, Claudio Belvedere and Alberto Leardini performed the data analysis. Giammarco Gardini and Carlo Capodagli wrote the manuscript. Cesar De Cesar Netto, Victor Valderrabano and Massimiliano Mosca supervised the analyses and revised the manuscript. All authors confirm that the article has never been published and is not under consideration for publication elsewhere. The paper has not been submitted to any other journal. All the authors meet the criteria for authorship. All the authors have read and approved the final manuscript and the data being presented in the manuscript and are fully responsible for it.

## FUNDING INFORMATION

The authors have no funding to report.

## CONFLICT OF INTEREST STATEMENT

Cesar De Cesar Netto: Paragon28 (consultant, medical advisory board, royalties), CurvebeamAI (consultant, shareholder), Ossio (consultant), Zimmer (consultant), Stryker (consultant), International WBCT Society (co‐founder, President), exactech (consultant), arthrex (consultant), tayco brace (shareholder), extremity medical (consultant), aofas committee member, foot ankle clinics (editor in chief). The remaining authors declare no conflicts of interest.

## ETHICS STATEMENT

The study was approved by local Ethics Committee AVEC:/SPER/IOR (n. 0007475), and the patient gave written informed consent in accordance with the Declaration of Helsinki. Informed consent was obtained pre‐analisys, following the Declaration of Helsinki, ensuring data confidentiality.

## Data Availability

The data that support the findings of this study are available from the corresponding author upon reasonable request. The data are not publicly available due to privacy and ethical restrictions related to patient confidentiality.
